# Depression, Anxiety, and Post-traumatic Stress Disorder Following a Hypertensive Disorder of Pregnancy: A Narrative Literature Review

**DOI:** 10.3389/fcvm.2019.00147

**Published:** 2019-10-09

**Authors:** Lynne Roberts, Greg K. Davis, Caroline S. E. Homer

**Affiliations:** ^1^Women's and Children's Health, St. George Hospital, Sydney, NSW, Australia; ^2^Faculty of Health, University of Technology, Sydney, NSW, Australia; ^3^St George and Sutherland Clinical School UNSW Medicine, University of New South Wales, Sydney, NSW, Australia; ^4^Maternal and Child Health Program, Burnet Institute, Melbourne, VIC, Australia

**Keywords:** depression, anxiety, post-traumatic stress disorder, postpartum, hypertensive disorder of pregnancy, gestational hypertension, preeclampsia

## Abstract

**Introduction:** Pregnancy and childbirth can be a source of anxiety and worry for women. This is probably more so for women with a complicated pregnancy. Anxiety and worry may contribute to, or exacerbate, mental health disorders including depression and post-traumatic stress disorder (PTSD). Mental health is an integral part of health and well-being and poor mental health can be detrimental to the woman's welfare and her infant's behavior and cognitive development. It may be undetected, potentially leading to a burden on the woman, her family, the health system, and society. Women with complicated pregnancies, such as those with hypertensive disorders of pregnancy (HDP), may be at greater risk of poor mental health. The aim of this review was to examine whether there is an association between depression, anxiety, and PTSD in postpartum women with a history of HDP.

**Methods:** A narrative literature review was undertaken. Using the key search terms: preeclampsia, gestational hypertension, hypertensive disorders, pregnancy complications, depression, anxiety, and post-traumatic stress disorder; electronic databases were searched to determine what is known about depression, anxiety, and PTSD after HDP.

**Results:** In total, 17 publications were included. The relationship between HDP and depression, anxiety, and PTSD was variable between studies and inconsistent. Although some studies reported no significant association, there is a trend for increased prevalence and symptom severity of depression, anxiety, and PTSD following HDP. This trend was particularly evident following the more severe presentations of HDP. It was uncertain whether this association was due to the hypertensive disorder itself, the sequelae of the HDP, such as giving birth to a preterm baby, or it predated the pregnancy.

**Conclusions:** Women who experience HDP may be at increased risk of developing postpartum depression, anxiety, and PTSD. Awareness of, and screening for, these mental health disorders in the postpartum period will alert clinicians to the need for additional follow-up and referral for women following HDP. More research on the benefits and risks of such an approach is needed.

## Introduction

Pregnancy and childbirth can be a source of stress and worry for many women (1) and mental health disorders following childbirth are common. Worldwide 10% of pregnant women and 13% of women who have just given birth experience a mental health disorder, primarily depression (2). These rates are higher (15.6 and 19.8%, respectively) in low to middle income countries (2). One in 7 women experience depression in the year following birth and one in five experience anxiety, commonly in combination with depression, during the same period (3). Research on the global prevalence of postpartum post-traumatic stress disorder (PTSD) is sparse and has been reported as 1–2% (4) following childbirth, although a literature review by Simpson and Catling (5), including papers from several countries, found that 20–48% of women reported their birth as a traumatic event which could potentially lead to PTSD.

Pregnancy and childbirth are likely to be more stressful for a woman experiencing a pregnancy with complications (6) and as a psychological manifestation, stress may coexist with depression, anxiety, and trauma reactions (7). A pregnancy may be considered complicated when there is an increased risk compared to a healthy pregnancy (1) and implies a threat to the woman's health, well-being, her baby, or both (8). An example of such a pregnancy is one complicated by a hypertensive disorder.

Hypertensive disorders of pregnancy (HDP) are common and complicate 10% of pregnancies (9) which equates to ~30,000 pregnancies a year in Australia and 13 million pregnancies a year globally and they are one of the leading causes of maternal and perinatal morbidity and mortality (10). The two pregnancy-specific disorders are gestational hypertension (GH) and preeclampsia (PE). GH is the new onset of hypertension after 20 weeks gestation (11) and when it presents at term, is usually a benign condition with little risk of adverse pregnancy outcomes (11, 12). However, the earlier GH presents in the pregnancy or the more severe the hypertension, the greater the risk of it progressing to PE or to an adverse pregnancy outcome (11, 13, 14). PE is a multi-system disorder which is described as the new onset of hypertension after 20 weeks gestation and the involvement of at least one other maternal organ system and/or the unborn baby (11, 12). Two particularly serious manifestations of PE are the syndrome HELLP (11, 12), comprising Hemolysis, Elevated Liver enzymes and a Low Platelet count, and eclampsia, which is seizures in a women with PE (11). There are long term physical health consequences for women after a HDP such as hypertension, stroke, heart attack, kidney disease, and diabetes (11, 15–19), however there is little known about women's mental health following this complication.

Poor mental health can impact both maternal and infant health negatively. In addition to affecting the woman's emotional welfare and everyday functioning, poor mental health may affect her parenting ability and can impair her relationship with her baby (20). Long term and/or untreated poor maternal mental health has been associated with poor infant well-being particularly in terms of behavioral and cognitive development (21). In severe cases, women with postpartum depression may commit suicide (2) and in those women with psychotic illnesses, the risk of infanticide, though rare, must be considered (2). Due to the serious consequences of poor mental health, early diagnosis, and treatment interventions are imperative for the health and well-being of the woman and her infant.

In the postpartum period, poor mental health is often undetected and untreated (22), potentially leading to a burden on the woman, her family, the health system and society. Guidelines highlight the importance of implementing interventions targeting women displaying the early signs and symptoms of poor mental health (23–25). However, it is not always known which women in the postpartum period would benefit from specific interventions. If women who experience HDP are identified as being at increased risk of poor mental health, targeted screening may be useful and lead to more timely referral and treatment initiation.

## Aim

The aim of this review was to examine whether there is an increased risk of depression, anxiety, and PTSD in postpartum women with a history of HDP.

## Methods

A narrative literature review was undertaken. This approach was used as it enabled a broad search that was able to draw conclusions about the topic and assist in identifying gaps or inconsistencies in the body of knowledge (26). Ethics approval was not required for this review.

### Search Strategy

A comprehensive search of the literature was undertaken using the electronic databases of EBM Reviews (Cochrane Database of Systematic Reviews), EMBASE, Ovid MEDLINE(R), CINAHL (Cumulative Index to Nursing and Allied Health), Maternity and Infant Care, PsycINFO, and Google Scholar. The key search terms used were: preeclampsia, gestational hypertension, hypertensive disorders, pregnancy complications, depression, anxiety, and post-traumatic stress disorder. All possible combinations and spellings of these key search terms were used. Additionally, the reference lists of identified papers were manually examined for further studies that may have been missed in the initial search.

**Figure 1:**
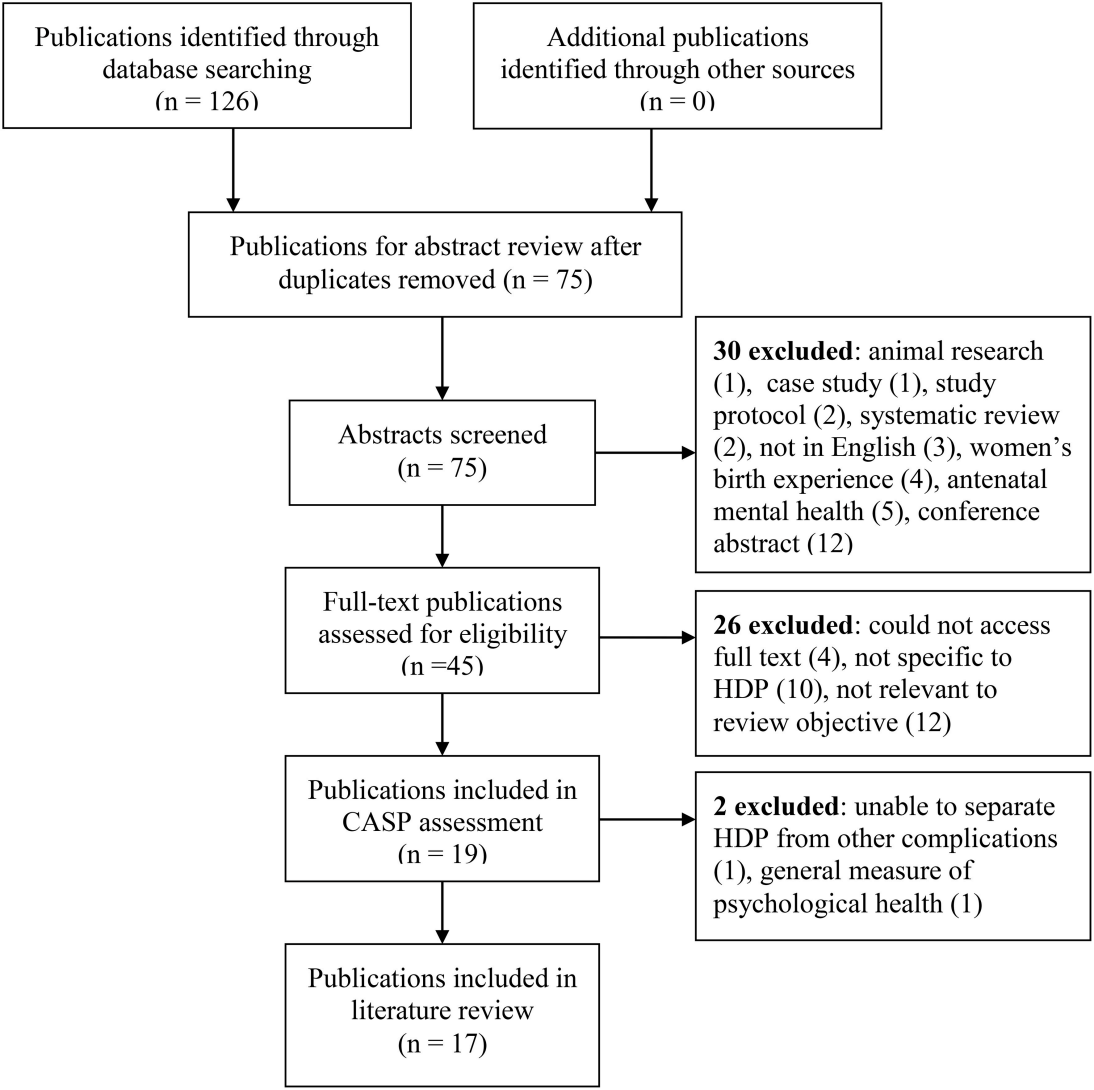
Publication selection.

The search was undertaken in May 2019, limited to primary publications published in English from the year 2000 onwards, with full text available. Publications only available in abstract form, conference abstracts, and study protocols were excluded as were systematic reviews (Figure 1). Two systematic reviews were excluded as there was a concern that including these would mean double counting of some studies. All relevant individual studies within these systematic reviews were included in this review. One systematic review (27) focussed on the relationship between PTSD and severe maternal morbidity. Several pregnancy complications were included with PE in four publications (28–31). The other systematic review (32) reported on depression and/or anxiety following a pregnancy complicated with severe PE and included six publications (28, 29, 31, 33–35).

Following title and abstract screening, 45 publications were identified for possible inclusion and the full text was sourced. Subsequent to full text reading, 19 were considered relevant to the search objective and were further assessed using the Critical Appraisal Skills Programme (CASP) (36). The CASP is a tool developed in 1993 which is widely used to systematically examine research articles to help identify the strengths, weaknesses, and usefulness of the study by appraising the validity, results and clinical relevance. Using the CASP checklist 17 publications were included in the review. Two publications were excluded at this stage as they: (1) reported on general quality of life following HDP and not specifically on depression, anxiety, or PTSD. Depression and anxiety are included in the screening instrument (SQL-90), but the overall results were reported as one score, so details specific to depression and anxiety could not be determined; and (2) reported the combined results of mental health for women after HDP and postpartum hemorrhage, making it impossible to separate the results for the HDP cohort.

## Results

In the included publications, the definition of HDP was sourced from different national and international guidelines (9, 37–39) (Table 1). Although these different sources were used, the definitions of the hypertensive disorders were consistent. However, the use of different study populations was not all directly comparable. For example, the severity of HDP varied, with some only including women with severe HDP and some only included women at a specific preterm gestation (Table 1).

**Table 1:** The definition of hypertension in pregnancy and inclusion criteria for the included studies.

**References**	**Source**	**Gestational hypertension**	**Preeclampsia**	
			**Mild**	**Severe**
Abedian et al. (40)	ACOG	Not eligible	At least BP ≥ 140/90 and proteinuria ≥ 30 mg/dl	
Baecke et al. (28)	National high blood pressure education program working group	Not eligible	BP ≥ 140/90 after 20 weeks gestation and proteinuria ≥ 300 mg/24 h Two groups: <37 weeks gestation ≥37 weeks gestation	
Blom et al. (33)	ISSHP	BP ≥ 140/90 after 20 weeks gestation	BP ≥ 140/90 after 20 weeks gestation and proteinuria ≥300 mg/24 h	
Brusse et al. (34)	ACOG	Not eligible	Not eligible	BP ≥ 140/90 and *de novo* proteinuria ≥300 mg/day and one or more of: • BP ≥160/110 mmHg on two occasions at least 6 h apart • Oliguria of <500 ml/24 h • Persistent headache or cerebral or visual disturbances • Epigastric pain • Increased serum creatinine level • Impaired liver function • Thrombocytopenia • Fetal growth restriction
Chen (41)	ACOG	Not eligible	Not stated	
Engelhard et al. (29)	National high blood pressure education program working group	Not eligible	Not stated Two groups: <37 weeks gestation ≥37 weeks gestation	
Fields et al. (42)	Not stated	Not eligible	Hospital adaptation of the International Classification of Disease codes used in record linkage	
Gaugler-Sedden et al. (35)	Not stated	Not eligible	Not eligible	DBP ≥ 110 mmHg and proteinuria 1 g/L, with or without • Eclampsia or HELLP (platelet count <100,000/μL, AST or ALT >70 U/L, lactate dehydrogenase >600 U/L) Two groups: <24 weeks gestation 24–32 weeks gestation
Habli et al. (43)	Not stated	Not eligible	Not eligible	HELLP—serum haptoglobin level <0.24 g/L and/or lactic dehydrogenase levels >600 U/L, AST>70 U/L, ALT >70 U/L, and platelet count ≤ 100,000/μL Two groups: <28 weeks gestation ≥28 weeks gestation
Hoedjes et al. (44) PND	ACOG	Not eligible	BP ≥ 140/90 and ≥300 mg/day proteinuria after 20 weeks gestation	At least one of: • BP ≥160/110 mmHg, • Proteinuria ≥5 g/day, • HELLP (platelet count <100,000/μL, AST >30 U/L, ALT >30 U/L), • Convulsion, • Fetal growth restriction
Hoedjes et al. (44) PTSD	ISSHP (mild PE) ACOG (severe PE)	Not eligible	BP ≥140/90 and ≥300 mg/day proteinuria after 20 weeks gestation	At least one of: • BP ≥160/110 mmHg, • Proteinuria ≥5 g/day, • HELLP (platelet count <100,000/μL, AST >30 U/L, ALT >30 U/L), • Convulsion, • Fetal growth restriction
Mautner et al. (45)	German guidelines	Not stated	Not stated	Not stated
Mommersteeg et al. (46)	ISSHP	Not eligible	DBP ≥90 with proteinuria (≥0.3 g/24 h) 20–32 weeks gestation	
Porcel et al. (47)	None	Self-reported	Self-reported	Self-reported
Postma et al. (48)	ISSHP	Not eligible	*De novo* hypertension after 20 weeks gestation plus proteinuria Eclampsia: new onset of seizures in women with preeclampsia Two groups: <34 weeks gestation ≥ 34 weeks gestation	
Postma et al. (48)	ISSHP	Not eligible	*De novo* hypertension after 20 weeks gestation plus proteinuria Eclampsia: new onset of seizures in women with preeclampsia	
Stramrood et al. (31)	ISSHP	Not eligible	Not stated	Not stated

*ALT, alanine aminotransferase; AST, aspartate aminotransaminase; BP, blood pressure; DBP, diastolic blood pressure; HELLP, Hemolysis, Elevated Liver enzymes, Low Platelets; PE, preeclampsia*.

The identified publications were grouped into three categories of mental health disorder, depression, anxiety, and PTSD. Some studies reported on more than one disorder and were included in more than one group. Furthermore, some studies reported on both prevalence and symptom severity. The results of depression, anxiety and PTSD were reported separately.

See Box 1 for a summary of each publication included in this review and Table 2 for a summary of the results.

Box 1 **Summary of papers selected for review**

Abedian et al. (40) was a descriptive study undertaken in Iran. Women diagnosed with PE (*n* = 100) were studied at 2 weeks and again at 6 weeks postpartum for depression, anxiety, and PTSD. The main focus of the paper was PTSD and the aim was to compare PTSD in primiparous (*n* = 56) and multiparous (*n* = 44) women.

Baecke et al. (28) was a cohort study undertaken in The Netherlands that included 170 women six-18 months postpartum, and measured depression, anxiety, and PTSD. Women with PE were included at term and preterm in addition to a normal comparison group who gave birth at term and preterm. The main focus of this study was cognitive function after PE.

Blom et al. (33) was a cohort study undertaken in The Netherlands that recruited 4,941 women over a 4 year period with various pregnancy complications; HDP contributing 254 to the cohort and 770 in the normal comparison group. Women were recruited in the antenatal period and depression was measured at 2 months postpartum.

Brusse et al. (34) was a small pilot study undertaken in The Netherlands that assessed 20 women three-7 months postpartum. Depression and anxiety were measured in 10 women with a history of severe PE and 10 women who had a history of an uncomplicated pregnancy formed the normal comparison group. The main focus of this study was cognitive function after PE.

Chen et al. (41) was a cohort study undertaken in China that examined depression at 6 week postpartum. The study cohort consisted of 90 women with a history of PE and 90 women, matched for the same time period of giving birth, who had normal blood pressure in pregnancy. The cohort was divided into two groups according to whether they screened positive for depression, and the analysis was performed comparing these two groups.

Englehard et al. (29) was a cohort study undertaken in The Netherlands that recruited 113 women within 2 years of hospital admission. Women with preterm and term PE were included and matched for gestational age with a normal comparison group. Depression and PTSD were measured. The women and their partners completed the screening instruments by post.

Fields et al. (42) used a medical data linkage to identify women for a long-term follow-up of cognitive impairment. Forty women who had a history of PE and 40 women with no such history were assessed 35–40 years following their pregnancy. Depression and anxiety formed part of the overall assessment but is reported separately.

Gaugler-Senden et al. (35) was a cohort study undertaken in The Netherlands that recruited 182 women who gave birth over an 11 year period. The cohort consisted of 104 women who had a history of preterm PE (<32 weeks gestation) and 78 women who had normal blood pressure in pregnancy, matched for gestational age at birth. Depression and PTSD was measured at two time points; women were asked to recall how they felt shortly after giving birth and the current time which was 7 years postpartum on average.

Habli et al. (43) was a retrospective cohort study undertaken in the US, using the US based HELLP Syndrome Support website to recruit 128 women. These women had experienced severe PE one to 31 years ago, with the mean follow-up being 5 years. Study designed surveys were completed via post, telephone or interview. Depression and anxiety prevalence was determined according to whether women reported being diagnosed by a physician with either of these mental health disorders.

Hoedjes et al. (44) was a cohort study undertaken in The Netherlands that recruited 174 women within 6 weeks after the birth. Depression was measured in women who experienced mild or severe PE at three time points: 6, 12, and 26 weeks postpartum. Comparisons were made between the two groups at each of the time points.

Hoedjes et al. (30) was a cohort study undertaken in The Netherlands, recruiting 149 women within 6 weeks after the birth. PTSD symptoms were measured in women who experienced mild or severe PE, at 6 and again at 12 weeks postpartum. Comparisons were made between the two groups at each of the time points.

Mautner et al. (45) was a cohort study undertaken in Germany that recruited 90 women with a complicated pregnancy, between 24 and 37 weeks gestation. Depression was measured during the pregnancy, 2-5 days and 3-4 months postpartum. The cohort consisted of women with HDP (*n* = 18), gestational diabetes (*n* = 11), preterm birth (32), and an uncomplicated pregnancy (*n* = 29). Comparisons were made between pregnancy groups at each of the time points.

Mommersteeg et al. (46) was a long term follow-up study conducted in The Netherlands examining depression and anxiety in women with a history of early onset PE (20–32 weeks gestation at onset), compared with a maternal aged matched non-PE comparison group. Questionnaires regarding depression and anxiety were completed, on average 14 years after the index pregnancy.

Porcel et al. (47) was a cross sectional online survey conducted via the worldwide Preeclampsia Foundation website. Women self-reported a diagnosis of HDP and were asked to invite friends and family who did not have a history of HDP to complete the survey, forming a normal comparison group. It was a large study with 1,076 women in the HDP group and 372 in the normal comparison group. The time between the index pregnancy and completion of the questionnaire is not stated.

Postma et al. (48) was an observational cohort study conducted in The Netherlands. It was a long-term follow-up of women already participating in a research project. There were 46 women with a history of eclampsia, 51 with a history of PE, and 48 women who had a normotensive pregnancy, matched for age. The average elapsed time since the index pregnancy was 7 years. The main focus of this study was neurocognitive function after PE and eclampsia, however depression and anxiety were assessed.

Postma et al. (49) was a retrospective cohort study undertaken in The Netherlands. Women with a history of eclampsia or PE were combined to form one group and the results were compared to women who were normotensive in their pregnancy. Although difficult to confirm, this study seems to have been an extension of the follow-up period in the above study. The cohort consisted of 41 women with prior eclampsia, 49 with a history of PE, and 47 women with no such history. The main focus of the study was cerebral white matter lesions and cognitive functioning following HDP, with depression and anxiety included in the assessment.

Stramrood et al. (31) was a prospective longitudinal cohort study undertaken in The Netherlands. The objective was to compare the prevalence and risk factors for PTSD in women with PE or preterm premature rupture of membranes (PPROM) compared to women with uncomplicated pregnancies. Women with PE were recruited in the antenatal period and depression and PTSD were measured at three time points: in pregnancy, 6 weeks and 15 months after the birth. The cohort consisted of women with PE or PPROM, and controls.

**Table 2:** Results of included studies reporting on HDP and postpartum depression, anxiety, or PTSD.

**References**	**Study design** **recruitment**	**Location**	**Instrument**	**When measured**	**Cohort (*N*)**	**Results**		
						**Depression**	**Anxiety**	**PTSD**
Abedian et al. (40)	Descriptive study	Iran	BDI STAI PPQ	2 weeks pp 6 weeks pp	PE (*n* = 100) 56 primiparous 44 multiparous	No significant difference at both time points (no figures given)	No significant difference at both time points (no figures given)	**Mean scores:** Primip 4.4 Multip 5.4 (*p* = 0.05) **Prevalence:** Primip 21% Multip 32% (*p* = 0.24) (unclear which time these results are from)
Baecke et al. (28)	Cohort study Recruited after birth	Netherlands 1 Hospital	BDI IES >25 STAI	6–18 months pp	Preterm PE (*n* = 48) Term PE (*n* = 18) PTB (*n* = 32) Term birth (*n* = 72)	**Mean scores:** Preterm PE 2.7 Term PE 1.3 PTB 1.2 Term birth 1.8 (*P* = 0.11)	**Mean scores:** Preterm PE 39.9 Term PE 39.6 PTB 35.4 Term birth 37.2 (*P* = 0.27)	**Prevalence:** Preterm PE 44% Term PE 11% PTB 41% Term birth 11% (*P* < 0.001) **Mean scores:** Preterm PE 25.3 Term PE 10.5 PTB 20.0 Term birth 12.7 (*P* < 0.001)
Blom, et al. (33)	Prospective cohort study Recruited antenatally	Netherlands Women in Rotterdam with EDC 04/2002-01/2006	EPDS > 12	2 months pp	PE (*n* = 183) Other (*n* = 3917) Normal (*n* = 770)	**Prevalence:** PE 16.9% GH 7.6% PE associated with increased risk of PND (OR 2.58, 95% CI 1.30–5.14)		
Brusse et al. (34)	Cohort study Recruited after birth	Netherlands 1 hospital	CES-D STAI	3–7 months pp	PE (*n* = 10) Normal (*n* = 10)	**Median scores:** PE 7.5 Normal 8 (*P* = 0.67)	**Median scores:** PE 39 Normal 31 (*P* = 0.21)	
Chen et al. (41)	Retrospective	China 1 hospital	EPDS ≥ 10	6 weeks pp	PE (*n* = 90) N (*n* = 90)	**Mean scores:** PE 7.27 N 4.42 (*p* = 0.05) **Prevalence:** PE 27% N 12% (*p* = 0.014)		
Engelhard et al. (29)	Cohort study Recruited after birth	Netherlands 1 Hospital	BDI>15 PSS	Within 2 years of hospital admission	Preterm PE (*n* = 18) PTB (*n* = 29) Term PE (*n* = 23) Term birth (*n* = 43)	**Prevalence:** Preterm PE 33% PTB 24% Term PE 26% Term birth 7%		**Prevalence:** Preterm PE 28% PTB 28% Term PE 17% Term birth 0%
Fields et al. (42)	Cohort study Women identified via medical record linkage	US	BDI BAI	Once, average 34.7 years since index pregnancy	PE (*n* = 40) N (*n* = 40)	**Mean scores:** PE 4 N 2 (*p* = 0.64)	**Mean scores:** PE 3 N 105 (*p* = 0.58)	
Gaugler-Senden et al. (35)	Cohort study Recruited after birth	Netherlands 1 hospital birthed 1993–2004	ZDS IES >19	In 2008 asked to recall symptoms in pp period and at current time	PE (*n* = 104) Normal (*n* = 78)	**Mean scores:** *Postpartum recall:* PE 11.78 N 11.37 (*P* = 0.59) *In 2008:* PE 5.4 Normal 5.51 *(P* = 0.87)		**Mean scores:** *Postpartum recall:* PE 29.72 Normal 27.53 (*P* = 0.41) *Currently:* PE 28.66 Normal 25.69 *(P* = 0.02)
Habli et al. (43)	Cohort study	US Advertised on HELLP Syndrome Society website	Study designed survey	1–31 years after birth	HELLP (*n* = 128)	**Prevalence:** 32%	**Prevalence:** 26%	
Hoedjes et al. (44)	Prospective cohort study Recruited within 6 weeks pp	Netherlands 4 hospitals	EPDS ≥ 10 EPDS ≥ 13	6 weeks pp 12 weeks pp 26 weeks pp	Mild PE (*n* = 39) Severe PE (*n* = 122)	**Prevalence:** (≥10) at any time: Mild PE 23.1% Severe PE 44.3% (*P* = 0.018) (≥13) at any time: Mild PE 15.4% Severe PE 26.2% (*P* = 0.165)		
Hoedjes et al. (30)	Prospective cohort study Recruited within 6 weeks pp	Netherlands 4 hospitals	Self-rating Inventory for PTSD	6 weeks pp 12 weeks pp	Mild PE (*n* = 35) Severe PE (*n* = 114)			**Prevalence total study population:** 6 weeks pp 8.6% 12 weeks pp 5.1% *(P* = 0.083)
Mautner et al. (45)	Prospective cohort study Recruited antenatally between 24 and 37 weeks gestation	Germany 1 tertiary referral hospital	EPDS ≥10	24–37 weeks gestation 2–5 days pp 3–4 months pp	HDP (*n* = 18) PTB (*n* = 32) Normal (*n* = 29)	**Mean scores:** 2–5 days pp HT 7.83 PTB 9.91 Normal 4.69 3–4 months HT 3.67		
			PTB 6.53 Normal 5.48			**Prevalence in HT:** 39% antenatally 27% 2–5 days pp 17% 3–4 months pp		
Mommersteeg et al. (46)	Cohort study	Netherlands 1 hospital	PHQ-9 ≥5 GAD-7 ≥5	Once, average 14 years after index pregnancy	PE (*n* = 265) N (*n* = 268)	**Prevalence:** PE 27% N 23% (*p* = 0.27) **Mean scores:** PE 3.6 N 2.9 (*p* = 0.03)	**Prevalence:** PE 31% N 27% (*p* = 0.33) **Mean scores:** PE 3.6 N 3.5 (*p* = 0.72)	
Porcel et al. (47)	Cross sectional online survey First pregnancy 1990–2010	Worldwide PE Foundation US based	BSSS for DSM IV ≥4	Once	HDP (*n* = 1,076) Normal (*n* = 372)			**Prevalence:** HDP 43% Normal 14% *(P* ≤ 0.01)
Postma et al. (48)	Observational cohort study Recruited 1–27 years after index pregnancy	Netherlands	HADS	Once, at least 12 months after the index pregnancy	Eclampsia (*n* = 46) PE (*n* = 51) Normal (*n* = 48)	**Mean scores:** Eclampsia 5 PE 4 Normal 3 (*P* = 0.02)	**Mean scores:** Eclampsia 7 PE 6 Normal 5 (*P* < 0.005)	
Postma et al. (49)	Retrospective cohort study	Netherlands	HADS	1–27 years since index pregnancy (mean = 6 years)	PE (*n* = 90) Normal (*n* = 47)	**Mean total scores:** PE 11 N 8 (*p* < 0.001)	**Mean total scores:** PE 11 N 8 (*p* < 0.001)	
Stramrood et al. (31)	Prospective cohort study Recruited antenatally when hospitalized (PE) or at 38 weeks gestation (controls)	Netherlands 1 hospital 1 midwifery practice	BDI > 20 PSSR-SR	Pregnancy 6 weeks pp 15 months pp	PE (*n* = 63) Normal (*n* = 65)	**Prevalence:** *Pregnancy* PE 19% Normal 7.7% *6 weeks pp* PE 10.5% Normal 6.2% *15 months pp* PE 6.8% Normal 0%		**Prevalence:** *6 weeks pp* PE 10.5% Normal 3% *15 months pp* PE 11.4% Normal 0%

*BDI, Beck Depression Inventory; BSSS, Breslau Short Screening Scale; CES-D, Center for Epidemiological Studies Depression Scale; CI, Confidence Interval; DSM IV, Diagnostic Statistical Manual of Mental Disorders 4th Edition; EDC, expected date of confinement; EPDS, Edinburgh Postnatal Depression Scale; GH' gestational hypertension; HADS, Hospital Anxiety and Depression Scale; HELLP, Hemolysis Elevated Liver enzymes Low Platelets; HDP, hypertensive disorder of pregnancy; IES, Impact of Event Scale; OR, Odds Ratio; PE, preeclampsia; PND, postnatal depression; pp, postpartum; PSS, Post-traumatic stress Symptom Scale; PSSR-SR, PTSD Symptom Scale self-report questionnaire; PTB, preterm birth; PTSD, post-traumatic stress disorder; SCL-90, 90 item Symptom Check List; STAI, State Trait Anxiety Inventory; ZDS, Zung Depression Scale*.

### Depression

Fifteen publications examined the association between HDP and depression (28, 29, 31, 33–35, 40–46, 48, 49). Eight reported on the prevalence of depression and 10 reported on symptom severity, using mean or median scores of the screening instrument used. Fourteen of these studies were observational cohort studies, and the other a descriptive design. The studies were conducted in The Netherlands (*n* = 10), United States (US) (*n* = 2), and one each in Iran, China, and Germany. There were seven different screening instruments used, and all bar one have been validated for accuracy and validity (see Table 3 for the screening instruments used).

**Table 3:** Summary of screening instruments for depression.

	**Design**	**Score**	**Validated**	**Cost**	**Comments**
BDI	21 questions Answer on feelings in past 2 weeks Scored on scale 0–3	>13 indicates depression Higher scores indicate greater depressive symptoms	Yes	Yes	Includes questions on tiredness and sleeping difficulties
CES-D	20 questions Answer on feelings in past week Scored on scale 0–3	>15 indicates depression Higher scores indicate greater depressive symptoms	Yes	No	Includes questions on tiredness and sleeping difficulties
EPDS	10 questions Answer on feelings in past week Scored on scale 0–3	Higher scores indicate greater depressive symptoms	Yes	No	Used widely through pregnancy and the postpartum period
HADS	14 questions Answer on feelings in past week Scored on scale 0–3	>10 indicates depression Higher scores indicate greater depressive symptoms	Yes	No	Mixture of questions regarding depression and anxiety. Depression and anxiety can be scored separately
PQH-9	9 questions Answer on feelings in past 2 weeks Scored on scale 0–3	>4 indicates depression Higher scores indicate greater depressive symptoms	Yes	No	Includes questions on difficultly sleeping and energy levels
ZDS	20 questions Scored on a scale of 1–4 Answer on feelings in ‘past several days’	Higher scores indicate greater depressive symptoms	Yes	No	Includes questions on difficultly sleeping and feeling tired

*BDI, Beck Depression Inventory (50); CES-D, Center for Epidemiological Studies Depression Scale (51); EPDS, Edinburgh Postnatal Depression Scale (52); HADS, Hospital Anxiety and Depression Scale (53), PQH-9, Patient Health Questionnaire (54); ZDS, Zung Depression Scale (55)*.

#### Prevalence of Depression

Reports of the prevalence of postpartum depression following HDP varied between 7 and 44% (29, 31, 33, 41, 43, 44, 46), which is up to four times higher than in the general postpartum population. This wide disparity in may be partly attributed to the inconsistencies in study design, particularly with regard to participant selection, severity of the hypertensive disorder, the screening instrument and the cut-off score used, and the timing of the evaluation.

When compared to women who did not have a history of HDP, a higher prevalence of depression was reported in women who experienced HDP (29, 31, 33, 45). It has been suggested that the psychological impact was influenced by the severity of the HDP, as characterized by gestational age at the onset, diagnosis of GH vs. PE, maternal complications, or adverse infant outcomes (29, 44). To investigate this more closely, Blom et al. (33) collected data from women following GH or PE separately and reported the prevalence of postpartum depression to be higher in the PE group compared with the GH group. Similarly, when data from women after mild or severe PE were analyzed separately, the prevalence of depression was higher in women with severe PE (33, 44). These results suggest that the prevalence of postpartum depression increases as the severity of the hypertensive disorder increases, however the results were not always statistically significant (44, 46).

A higher prevalence of depression was reported by women who gave birth preterm or term with PE when compared to women matched for gestation at birth with an otherwise uncomplicated pregnancy (29) suggesting that the PE contributed to the depression. In contrast, a Dutch study reported no difference in depression for women who gave birth preterm with or without PE (28), suggesting that maybe it was the consequences of a preterm birth rather than the HDP that was more strongly associated with depression.

When depression has been measured longitudinally at several postpartum time points following HDP, the prevalence was shown to decrease over time. A decrease from 27% at two-5 days postpartum to 17% at three-4 months postpartum was reported in one study (45) while a decrease from 10.5% at 6 weeks postpartum to 6.8% at 15 months postpartum was reported in another (31). However, there is no mention of any treatment that may have been undertaken during the period of the study, so it is unclear what contributed to the lessening depression.

#### Depression Symptom Severity

With regard to the severity of depressive symptoms, the evidence was mixed and inconclusive. Some studies report significantly higher mean scores on the screening instrument by women following HDP compared with women in the normal comparison group (41, 46, 48, 49). However, others report no significant difference in mean scores between women with or without prior HDP (28, 34, 35, 42), although there was a trend toward higher scores from women in the HDP group. Having a preterm birth, stillbirth, or neonatal death was significantly associated with higher depressive symptoms in one study (46) which reported on depression, on average, 14 years following the index pregnancy.

No significant differences in scores were reported comparing primiparous and multiparous women at 2 and 6 weeks postpartum following a pregnancy complicated with PE (40), although there were no details of the scores given. In another study, no significant difference in mean scores was found in four groups of women; preterm PE, term PE, preterm birth without HDP, and term birth without HDP (*p* = 0.11) (28). In a study examining depression following eclampsia and PE, statistically significant higher mean scores were reported by women who had experienced eclampsia compared to those women with a history of PE and those who were normotensive in pregnancy (*p* = 0.02) (48).

Mean scores between women with a history of preterm severe PE and preterm birth without PE at two time points postpartum reported no statistical difference in scores between the two groups (36), but notes that the mean scores improved with time (36). Women in this study were asked to recall and score symptoms from the immediate postpartum period for the first time point, with an average elapsed time of 7 years since the index pregnancy for the second time point.

### Anxiety

There were eight studies that investigated the association between HDP and anxiety (28, 34, 40, 42, 43, 46, 48, 49). Prevalence of anxiety was reported in six of these studies and symptom severity in seven using mean or median scores. Seven of the studies were observational cohort studies and one was a descriptive study. The studies originated from three different countries; The Netherlands (*n* = 5), US (*n* = 2), and Iran (*n* = 1). There were five different screening instruments used, and all except one have been validated for accuracy and validity (Table 4).

**Table 4:** Summary of screening instruments for anxiety.

	**Design**	**Score**	**Validated**	**Cost**	**Comments**
BAI	21 questions Answered on feelings in past week Scored on scale 0–3	0–21 low anxiety 22–35 moderate anxiety >35 potentially concerning levels of anxiety	Yes	Yes	Designed to minimize the overlap between depression and anxiety
GAD-7	7 questions Answer on feelings in past 2 weeks Scored on scale 0–3	0–5 mild anxiety 6–10 moderate anxiety 11–21 severe anxiety	Yes	No	Concludes with extra question about the impact on everyday activities
HADS	14 questions Answer on feelings in past week Scored on scale 0–3	>10 indicates anxiety Higher scores indicate greater anxiety	Yes	No	Mixture of questions regarding depression and anxiety. Depression and anxiety can be scored separately
STAI	40 questions (20 on state anxiety, 20 on trait anxiety) 4 point Likert Scale	State and trait anxiety scored separately. Scores range from 20 to 80 in each sub-scale >40 clinically significant. Higher scores indicate greater anxiety	Yes	Yes	Used widely in research Lengthy questionnaire requiring 15–20 min to complete

*BAI, Beck Anxiety Inventory (56); GAD-7, General Anxiety Disorder scale (57); HADS, Hospital Anxiety and Depression Scale (53); STAI, State Trait Anxiety Inventory (58)*.

#### Prevalence of Anxiety

The prevalence of anxiety following HDP was reported between 26 and 32% in two publications (43, 46), which is slightly higher than the general postpartum population. Both of these studies were long term follow-up studies with assessment of anxiety undertaken up to 31 years postpartum.

There was no difference in the prevalence of mild anxiety in women who had experienced early onset PE compared to women without a history of PE (46), with a reported prevalence of 31 vs. 27%, respectively (*p* = 0.325). The average time elapsed since the index pregnancy in this study was 14 years. On further analysis, adjusting for age, education level, body mass index (BMI), having a partner, being unemployed, and physical activity, the results showed no difference in the prevalence of anxiety between the two groups.

The prevalence of anxiety in women who experienced HELLP syndrome was reported as 26% (43). In this study anxiety was measured by women within 1 month of the birth, self-reporting a diagnosis made by a physician and treated accordingly. The gestation at the time of birth was not important, with 27% of those who gave birth at 28 weeks gestation or less and 26% of those women giving birth after 28 weeks gestation, reporting a diagnosis of postpartum anxiety (*p* > 0.99).

#### Anxiety Symptom Severity

Seven studies reported on anxiety symptom severity using mean or median scores derived from a validated instrument (28, 34, 40, 42, 46, 48, 49). Higher scores were reported from women in the HDP group when compared with women in the normal comparison group, however the majority of studies did not reach statistical significance. The two studies that reported statistically significant higher anxiety scores in the PE group compared to the comparison group (48, 49) were small studies where women completed the questionnaire, on average, 6 years after their pregnancy. The main focus of both these studies was cognitive functioning after a pregnancy complicated with PE, and there was no reporting of any other factors or life events that may have affected the woman's anxiety.

No significant difference in anxiety scores was reported by primiparous compared with multiparous women at both the 2 and 6 week postpartum following a pregnancy complicated with PE (40), although there were no details of the scores given in the publication. No significant difference in mean scores was found in the four groups studied by Baecke et al. (28). These groups were women who experienced preterm PE, term PE, preterm birth without HDP, and term birth without HDP (*p* = 0.27). In a study examining anxiety following eclampsia and PE, statistically significant higher mean scores were reported by women who had experienced eclampsia compared to those women with a history of PE and those who were normotensive in pregnancy (*p* < 0.005) (48), suggesting that the more severe the HDP, the more severe the anxiety is.

### Post-traumatic Stress Disorder

There were seven studies that investigated the association of PTSD with HDP (28–31, 35, 40, 59), with six reporting on prevalence and three reporting on symptom severity by using mean scores for the instruments used. There were five observational cohort studies, one descriptive study and one cross-sectional on-line survey. The studies were conducted in The Netherlands (*n* = 5), Iran (*n* = 1), and the remaining one was a world-wide survey led by researchers in the US. All studies used validated instruments to screen for PTSD and there were five different instruments used (Table 5).

**Table 5:** Summary of screening instruments for PTSD.

	**Design**	**Score**	**Validated**	**Cost**	**Comments**
BSSS	7 yes/no questions Scored 1 for ‘yes’, 0 for ‘no’ Answered on feelings in past month	>4 PTSD	Yes	No	Measures avoidance and numbing, hyperarousal
IES	22 items Scored on scale 0–4 Answered on feelings in past week	24–32 some symptoms 33–37 probable diagnosis of PTSD >37 PTSD	Yes	No	Measures intrusions, avoidance, and numbing, hyperarousal
PPQ	14 items Scored on scale 0–4	>18 PTSD	Yes	No	Designed to measure PTSD symptoms related to childbirth and symptoms during postnatal period Measures intrusions, re-experiencing, avoidance, and numbing, hyperarousal
PSS	17 items Scored on a scale 0–3	13 and over likely PTSD	Yes	Yes	Measures re-experiencing, avoidance, and arousal Based on DSM-IV
SRI for PTSD	22 items Scored on a scale 1–4	>51 PTSD	Yes		Based on DSM-IV

*BSSS, Breslau Short Screening Scale (60); IES, Impact of Event Scale (61); PPQ, Perinatal Post-traumatic Stress Questionnaire (62); PSS, Post-traumatic stress Symptom Scale (63); SRI for PTSD, Self-rating inventory for PTSD (64)*.

#### Prevalence of PTSD

The prevalence of PTSD following HDP varied between 5.1 and 43% across the seven studies. When compared to women who did not have a history of HDP, a higher prevalence of PTSD was reported in women who experienced HDP in some of the studies (29, 31, 47). In the world wide survey via the PE Foundation website (47), 43% of women with a history of HDP screened positive for PTSD compared to 14% of women in the normal comparison group (*p* < 0.01). In this study, after adjusting for psychiatric treatment, parity, and age at the time of the pregnancy, women with a history of PE were more than four times as likely to screen positive for PTSD when compared to women with a normotensive pregnancy (OR = 4.46, 95% CI: 3.20–6.20).

The study by Stramrood et al. (31) measured PTSD at two postpartum time points (6 weeks and 15 months) and compared results between women in the PE and normotensive groups at each of these time points. There was a slight change in prevalence over time in both groups (10.5–11.4% in the PE group and 3–0% in the normotensive group), with the PE group consistently reporting a higher prevalence of PTSD. The other study to report PTSD prevalence at two time points (30), found no statistically significant difference in PTSD in women with a history of PE, at 6 and 12 weeks postpartum, with rates of 8.6 and 5.1%, respectively (*p* = 0.083).

Irrespective of the cause of the preterm birth, Baecke et al. (28) found that more women with a history of a preterm birth met the threshold score for PTSD than women who gave birth at term (preterm PE 44%, preterm birth 41%, term PE 11%, term uneventful 11%). This study suggests that the sequelae of a preterm birth led to PTSD, not the PE itself. In the Dutch study by Engelhard et al. (29), 28% of women who gave birth preterm, with or without PE, met the diagnostic criteria for PTSD, suggesting that the preterm birth rather than PE is the trigger. However, in this same study, the term PE group reported a PTSD prevalence of 17%, similar to that in preterm PTSD, while the normotensive term group women had no PTSD, suggesting that PE may have an impact at term.

A further study compared PTSD prevalence in multiparous and primiparous women with a history of PE (40). Although the reported PTSD prevalence was higher in the multiparous PE group, the result was not significant (32 vs. 21%, respectively *p* = 0.24) suggesting that parity is not a contributing factor.

#### PTSD Symptom Severity

There were three studies that reported on PTSD symptom severity following PE (28, 35, 40), all with different study designs and all using a validated instrument to score PTSD symptoms.

When mean scores were compared between primiparous and multiparous women following PE, the results were not statistically significant although the multiparous women scored slightly higher (mean score 4.4 for primiparous women and 5.4 for multiparous women *p* = 0.05) (40).

The mean PTSD scores for women with a history of preterm birth, irrespective of the cause, were higher than the term birth group, suggesting a preterm birth contributes to more symptom severity (28).

Another study (35) compared mean PTSD scores between women with a history of preterm severe PE and preterm birth without PE at two time points. There was no difference in mean scores between the groups at the postpartum recall time-point. However, there was a significant difference (*p* = 0.02) between the groups at the time of the study being undertaken which was, on average, 7 years after the index pregnancy, despite the mean score being slightly lower. There is no information about other life events that may have contributed to this difference.

## Discussion

There is limited literature available to address the important issue of depression, anxiety, and PTSD following pregnancies complicated by hypertension with only 17 studies identified that looked specifically at this association. The current evidence suggests that women with a history of HDP have a greater likelihood of depression and PTSD, but the heterogeneity, different study populations and different methods of assessment preclude any definitive interpretation. There was no association found between HDP and the prevalence or severity of postpartum anxiety in the majority of studies included in this review. The current literature also suggests that the severity of the HDP may be positively correlated with the severity of depressive, anxiety and PTSD symptoms. However, it is not clear what the main driver for the psychological morbidity might be—the demands of PE, the associated events such as a preterm birth or if it predated the pregnancy. The focus of this review was on postpartum mental health conditions, hence pre-existing and/or antenatal mental conditions were not investigated.

The literature on the psychological impact of HDP was methodologically varied, including selection and recall bias and most study sample sizes were small. There was a variation in the study populations including gestation, severity of HDP and time since the index pregnancy. Furthermore, multiple instruments were used, along with different threshold cut-off scores to define abnormal results. These methodological limitations made it difficult to determine if there is a true association between HDP and depression, anxiety and PTSD.

A key feature with the studies included is that they originate from just five countries; 11 from The Netherlands, three from the US, and one each from China, Germany, and Iran. It may not be possible to accurately translate the results of these studies to other countries, due to the differences in culture, health care systems, and management of HDP and/or mental health disorders. The performance of the measuring instruments may also be affected by cultural practices, differences in access and availability of, obstetric and psychiatric care and differences in emotional support provided. Some researchers (65, 66) suggest that culture affects the response people make to psychiatric assessments due to the differences in their underlying attitudes, beliefs, and behavior. Beliefs of mental health distress, cultural understanding of mental health, and culturally or context specific terms can also lead to different scores on measuring instruments (67).

Previous reviews of women's mental health following HDP have made similar conclusions to those drawn here. A systematic review (32) reported on postpartum depression, anxiety, and PTSD following a pregnancy complicated with HDP. The authors conclude that the evidence regarding depression is mixed but overall suggests an association between PE/HELLP and depression, with higher depression prevalence and severity in the women with previous PE/HELLP compared to women without such history. In regards to anxiety, there were no significant associations between PE and anxiety scores although higher scores were reported among women with PE. PTSD was reported in this same review and although higher PTSD prevalence and severity was reported by women following PE/HELLP, results were not statistically significant. In another systematic review (27) investigating the possible association between PTSD and several pregnancy complications, including PE, the authors conclude that there may be some evidence to suggest a link between PE and PTSD but the evidence was not robust (27). However, they suggest that PTSD and its symptoms may present following particularly severe cases of maternal morbidity (not specifically HDP), that involve poor neonatal outcomes.

## Conclusion

While there is no definitive evidence that having HDP leads to increased postpartum depression, anxiety, or PTSD, women who experience HDP may be at increased risk of developing these mental health disorders. This is particularly true for those women who experience the more severe forms of HDP and/or give birth preterm. Routine screening for all these mental health disorders on all women in the postpartum period may be beneficial, however there is an increased need for screening to be undertaken in women who experience HDP. Screening this group of high risk women will alert clinicians to the need for additional follow-up and referral. More research on the benefits and risks of such an approach is needed.

## Author Contributions

LR, GD, and CH contributed to the conception and design of the review. LR led the review of the literature, the analysis, and wrote the first draft including drafting the tables and figure. All authors contributed to manuscripts drafts and revisions, and all approved the final submitted version.

### Conflict of Interest

The authors declare that the research was conducted in the absence of any commercial or financial relationships that could be construed as a potential conflict of interest.
